# Elevated Calprotectin Levels Reveal Loss of Vascular Pattern and Atrophy of Villi in Ileum by Digital Chromoendoscopy and Magnification Colonoscopy in Patients with Spondyloarthritis Without Having Inflammatory Bowel Disease †

**DOI:** 10.3390/diagnostics14222591

**Published:** 2024-11-18

**Authors:** Juliette De Avila, Cristian Flórez-Sarmiento, Viviana Parra-Izquierdo, Wilson Bautista-Molano, Magaly Chamorro-Melo, Adriana Beltrán-Ostos, Diego Alejandro Jaimes, Valery Khoury, Lorena Chila-Moreno, Alejandro Ramos-Casallas, Juan Manuel Bello-Gualtero, Jaiber Gutiérrez, Cesar Pacheco-Tena, Philippe Selim Chalem Choueka, Consuelo Romero-Sánchez

**Affiliations:** 1 Cellular and Molecular Immunology Group–InmuBo, School of Dentistry, Universidad El Bosque, Av. Cra 9 No. 131 A–02, Bogotá 110121, Colombia; dequiroga@unbosque.edu.co (J.D.A.); cristianfflorez@hotmail.com (C.F.-S.); dravivianaparra@gmail.com (V.P.-I.); wbautistam@unbosque.edu.co (W.B.-M.); abeltraninvestigaciones@gmail.com (A.B.-O.); lchila@unbosque.edu.co (L.C.-M.); maramos@unbosque.edu.co (A.R.-C.); 2 Gastroadvanced, Bogotá 110221, Colombia; 3 Clinical Immunology Group, Rheumatology and Immunology Department, School of Medicine, Hospital Militar Central, Universidad Militar Nueva Granada, Transversal 3ª # 49-00, Bogotá 110231, Colombia; yanni_magali1@hotmail.com (M.C.-M.); juanmabello36@gmail.com (J.M.B.-G.); jaibergutierrez1@gmail.com (J.G.); 4 Clínicos IPS, Bogotá 110231, Colombia; 5 School of Medicine, Universidad El Bosque, Bogotá 110121, Colombia; vkhoury@unbosque.edu.co; 6 Investigación y Biomedicina de Chihuahua, Chihuahua 31205, Mexico; dr.cesarpacheco@gmail.com; 7 Fundación Instituto de Reumatología Fernando Chalem, Bogotá 111211, Colombia; p_chalem@yahoo.com

**Keywords:** fecal calprotectin, spondyloarthritis, digital chromo-endoscopy, inflammatory bowel disease, microscopic inflammation

## Abstract

Objective: This study aimed to establish a correlation between fecal calprotectin levels (FC) and intestinal inflammation in patients with spondyloarthritis without inflammatory bowel disease. Methods: A total of 180 SpA patients were included in the study of them 20.6% required Digital chromoendoscopy (DCE). FC, C-reactive protein (CRP), HLA-B*27 and clinical indices were assessed. Results: Positive fecal calprotectin (PFC) and high fecal calprotectin (HFC) levels were observed in 27.0% and 16.0% of patients, respectively. HFC correlated with a Bath Ankylosing Spondylitis Functional Index (BASFI) score > 4.0 (*p* = 0.036) and a Bath Ankylosing Spondylitis Disease Activity Index (BASDAI) score > 4.0 (*p* = 0.047). Loss of vascular pattern in the ileum (LVPI) was observed in approximately 70.0% of patients (*p* = 0.005), which was associated with PFC and abdominal bloating (*p* = 0.020). LVPI was also linked to microscopic inflammation (*p* = 0.012) and PFC with abdominal pain (*p* = 0.007). HFC was significantly associated with alterations in the ileal mucosa (*p* = 0.009) and LVPI (*p* = 0.001). Additionally, HFC and diarrhea were associated with LVPI in 27.3% of patients (*p* = 0.037) and with erosions in the ileum (*p* = 0.031). Chronic ileal inflammation correlated with HFC (*p* = 0.015), ASDAS-CRP > 2.1 (*p* = 0.09), LVPI (*p* = 0.001), and villous atrophy (*p* = 0.014). Factorial analysis of mixed data (FAMD) identified significant associations between micro/macroscopic changes in chronic inflammation and HFC (CC = 0.837); increased levels of CRP and microscopic acute inflammation (CC = 0.792); and clinical activity scores of ASDAS-CRP and BASDAI (CC = 0.914). Conlusions: FC levels were significantly elevated in patients with SpA, particularly those with LVPI, suggesting their potential as a valuable biomarker for managing SpA when joint manifestations coincide with ileal villous atrophy. This indicates a shared immune pathway linked to chronic gut damage.

## 1. Introduction

Spondyloarthritis (SpA) constitutes a group of chronic autoinflammatory diseases predominantly affecting young men, presenting with either axial or peripheral manifestations. These conditions are characterized by progressive new bone formation leading to ankylosis and functional disability. Ankylosing spondylitis (AS) serves as a prototypic model for studying SpA, and these diseases share common clinical, radiographic, and immunogenetic features, notably the association with the histocompatibility molecule HLA-B*27, which influences disease severity [1,2,3].

Studies indicate that a proportion of 5–10% of patients diagnosed with AS may concurrently present with inflammatory bowel disease (IBD); conversely, over 60% of individuals devoid of IBD could demonstrate subclinical characteristics indicative of intestinal inflammation [4,5,6]. Moreover, the existing evidence suggests that a reduction in articular manifestations may occur when intestinal inflammation is adequately controlled in these individuals [4]. Ileocolonoscopy is regarded as the gold standard methodology for assessing intestinal inflammation, whereas dynamic contrast enhancement (DCE) combined with narrow-band imaging (NBI) and high magnification provides a robust endoscopic approach for the detection of both macroscopic and microscopic inflammation. This diagnostic approach holds promise for revealing subclinical intestinal inflammation in patients with SpA, particularly among those who exhibit nonspecific gastrointestinal symptoms [4,5,6,7].

In addition to endoscopic methodologies, fecal calprotectin (FC) has emerged as a salient biomarker indicative of intestinal involvement. FC, a calcium-binding protein derived from leukocytes, is acknowledged as a damage-associated molecular pattern (DAMP) that facilitates inflammatory responses [8,9,10]. Currently, calprotectin is recognized for its role in activating TLR-4 across various cellular phenotypes, which encompass neutrophils, macrophages, lymphocytes, and endothelial cells [11,12]. Consequently, FC may function as a sensitive and predictive biomarker for the surveillance of intestinal inflammatory activity across diverse pathological conditions, including SpA [12]. FC presents distinct advantages in differentiating organic diseases from functional disorders when compared to conventional markers such as C-reactive protein (CRP) and erythrocyte sedimentation rate (ESR). FC holds substantial significance in forecasting postoperative IBD recurrence, clinical relapse, and the achievement of mucosal healing [13,14,15].

A study by Cypers et al. evaluated serum calprotectin and ileocolonoscopy, incorporating FC assessment in a cohort of 44 subjects [16]. The results indicated that 42% of individuals diagnosed with the condition displayed microscopic inflammation of the intestines along with significantly elevated levels of FC. Alternatively, within the cohort of patients with heightened serum calprotectin and CRP levels, 64% presented with intestinal inflammation. Another investigation conducted by Turina MC posited that increased serum calprotectin concentrations, irrespective of radiographic advancement in SpA, could function as a prognostic marker for microscopic intestinal inflammation, thereby facilitating patient selection for ileocolonoscopy [17]. Some investigations carried out within the Colombian population have revealed the occurrence of gastrointestinal manifestations, the presence of autoantibodies, and heightened levels of FC in individuals diagnosed with active SpA without IBD [18]. The elevated concentrations of FC exhibited a notable association with systemic inflammatory markers, including CRP and ESR [18,19]. These findings thereby raise an inquiry regarding the potential relationship between FC levels and both macroscopic and microscopic alterations present within the intestinal tract. This finding might establish this DAMP as a promising noninvasive biomarker for the evaluation of intestinal involvement in individuals with SpA.

The assessment of specific clinical manifestations and gastrointestinal biomarkers may enhance the identification of patients requiring endoscopic and histological evaluation. Even in situations lacking clinical indicators particular to IBD, an endoscopic study combined with elevated FC levels is advised and could benefit patients with SpA. The objective of this study is to investigate the correlation and clinical significance of FC concentrations in conjunction with DCE magnification colonoscopy and histological evaluation for the early identification of intestinal inflammation in patients with SpA devoid of IBD.

## 2. Materials and Methods

### 2.1. Study Design and Population

A total of 180 adult patients (≥18 years old) with SpA, as defined by the European Spondyloarthropathy Study Group (ESSG) [20] and the Assessment of SpondyloArthritis International Society (ASAS) classification criteria [21,22], were assessed by rheumatologists at the outpatient rheumatology services clinic at the Hospital Militar Central and Clinicos IPS in Bogotá, Colombia.

A cross-sectional study was conducted using a convenience non-probability sampling method, acknowledging the challenges in recruiting patients who met the strict criteria for inclusion in the study. This limitation reflects the real-world difficulty in enrolling SpA patients with specific gastrointestinal symptoms who are willing and able to undergo extensive diagnostic testing, including endoscopy and histological analysis.

Exclusion criteria included pregnancy, breastfeeding, malignancies, autoimmune or autoinflammatory diseases, chronic inflammatory liver disease, pancreatitis, immunodeficiencies, antibiotic treatment within the past three months, and concomitant IBD.

All patients were evaluated for the presence of clinical symptoms, with medical records reviewed for gastrointestinal symptoms such as diarrhea (defined as more than three bowel movements per day), stools with mucus, hematochezia, number of stools per day, abdominal pain, abdominal bloating, food intolerances, and weight loss. SpA patients with two or more gastrointestinal symptoms were referred to the GastroAdvanced IPS/gastroenterology service. Among the 180 patients assessed, 42.2% (76/180) had an indication for gastroenterologist evaluation, and of these, only 20.6% (37/180) required chromo-endoscopy/digital colonoscopy and histological analysis.

Clinical activity indices such as BASDAI, ASDAS, and the BASFI functional index were collected for all patients [22,23,24,25]. Although there are no validated scores for assessing disease activity and functionality in peripheral SpA [26], these outcome measures are widely used to evaluate patients with Peripheral SpA (pSpA) and were included in this study.

### 2.2. Determination of Fecal Calprotectin-FC

Following collection, all fecal samples were stored at −20 °C to preserve their integrity until analysis. The concentration of calprotectin was measured in the supernatant of a centrifuged solution derived from 0.2 g of stool. This measurement was performed using a quantitative enzyme-linked immunosorbent assay (ELISA), in accordance with the manufacturer’s instructions for the Quantitative Fecal Calprotectin KAPEPKT849^®^ kit, DIASource (Rue du Bosquet 2, 1348 Louvain-la-Neuve, Belgium). The assay was conducted in a controlled laboratory environment, with all necessary quality controls in place. A cutoff value of >120 ng/mL was established for normal levels, while results > 250 ng/mL were classified as high levels (HFC) of FC, indicating potential inflammatory processes in the gastrointestinal tract. All samples were analyzed in duplicate to ensure accuracy and reliability of the results.

### 2.3. Determination of High-Sensitivity C Reactive Protein-CRP

The determination of high-sensitivity (hs-CRP) was performed using the Immulite 1000 (Siemens Medical Solution Diagnostics, Los Angeles, CA, USA) with the LKCRP1 kit. The assay was conducted following the manufacturer’s instructions, which included specific sample preparation, calibration, and quality control measures. Blood samples were collected from participants and allowed to clot before centrifugation at 2500 rpm for 10 min to obtain serum. The serum was then diluted as necessary, and the assay was conducted on the Immulite 1000 analyzer. Reference values for hs-CRP were established at 0–3 mg/dL, which are crucial for interpreting the results within the context of inflammatory disease assessment.

### 2.4. Determination of Erythrocyte Sedimentation Rate-ESR

The determination of (ESR) was performed according to the Wintrobe method for quantitative analysis. Blood samples were collected from participants using sterile techniques and were allowed to stand undisturbed for one hour in a vertical position. The sedimentation of erythrocytes was measured in millimeters per hour, following the manufacturer’s instructions. Specifically, the sample was placed in a Wintrobe tube, and the distance that the red blood cells settled was recorded after one hour. Values greater than 20 mm/h were considered elevated and indicative of potential inflammatory processes. All measurements were conducted in duplicate to ensure accuracy and reproducibility.

### 2.5. HLA-B27 Measurement

The measurement of HLA-B27 was conducted using next-generation sequencing (NGS) technology, specifically utilizing the Illumina/PacBio sequencing platforms (1× High Resolution Typing, HistoGenetics—The Genomics Company, Ossining, NY, USA). This involved the analysis of the second and third exons of the HLA-B gene to identify the presence of the HLA-B27 antigen. Sample preparation included DNA extraction from peripheral blood lymphocytes, followed by library preparation according to the manufacturer’s instructions. Sequencing was performed under optimized conditions to ensure high fidelity and accuracy. Bioinformatics analysis was subsequently conducted to interpret the sequencing data, allowing for the identification and quantification of the HLA-B27 allele in each sample. Results were validated against established reference sequences to confirm the presence or absence of the HLA-B27 antigen. The datasets presented in this study can be found in online repositories. The names of the repository/repositories and accession number(s) can be found below: https://www.ncbi.nlm.nih.gov/, PRJNA843059.

### 2.6. Colonoscopy by Digital Chromoendoscopy (DCE) with Narrow Band Imaging (NBI)

A clinical assessment was conducted by the Gastroenterology service, where all patients meeting the inclusion criteria were invited to participate in the study. The clinician provided a detailed explanation of the procedure, including its importance, potential benefits, and associated risks. Patients who consented to participate signed an informed consent form.

Prior to the colonoscopy, all participants underwent necessary preparation (Travad Pik, Technoquimicas Cali, CO, USA) to ensure optimal visualization during the procedure. The adequacy of colonic cleansing and the distal ileal passage was assessed using the Boston Bowel Preparation Scale (score of 9/9), indicating a high level of cleanliness.

Colonoscopy was performed by a gastroenterologist specialized in diagnostic and therapeutic endoscopy, utilizing sedation administered through an intravenous protocol with assistance from the anesthesiology team. Both submucosal vascular patterns and stromal alterations were evaluated in the villi of the ileum as well as in the crypt patterns of the colon. This assessment was achieved through electronic magnification of the mucosa, utilizing DCE with narrow-band imaging (NBI) (Olympus, Shinjuku City, Tokyo, Japan) and blue light imaging (BLI) (FUJI, Minato City, Tokyo, Japan). The procedures were carried out using Olympus EVIS EXERA III (CF-HQ190 L/I) and FUJI EC-760ZP-V/L Zoom, ELUXEO Series 700 equipment. 

To enhance visualization, irrigation with the endoscopic irrigation pump (EIP) was performed according to the established protocol and mucosal biopsies were obtained using a radial Max 4 Standard Capacity device (Boston Scientific, Alpharetta, GA, USA).

### 2.7. Histological Evaluation

The histological evaluation was conducted using paraffin embedding, fine cutting, and routine staining with hematoxylin-eosin. Tissue samples from the distal ileum, sigmoid colon, and rectum were analyzed to assess both acute and chronic inflammatory changes. Acute inflammation was evaluated based on the presence of polymorphonuclear neutrophils, cryptitis, cryptic abscesses, ulcers, gut fissures, and erosions. Chronic inflammatory changes were identified through architectural alterations and metaplastic and/or stromal changes, as well as immune infiltrates characterized by lymphocyte predominance and eosinophilia.

An average of 18 to 24 levels per slide was obtained from each sample to ensure comprehensive assessment. Special stains were applied as necessary, including Masson’s trichrome, Gomori’s methenamine silver (GMS), periodic acid-Schiff (PAS) with and without diastase, and Ziehl-Neelsen. Immunohistochemistry was also performed when indicated to provide further characterization of tissue alterations [27].

### 2.8. Statistical Analysis

A descriptive analysis was conducted for clinical, laboratory, and demographic data. Additionally, measures of central tendency and dispersion were calculated for clinical variables. The association between FC levels and DCE/histological findings was evaluated using the Chi-square test or Fisher’s exact test as appropriate.

A confirmatory multivariate evaluation was performed using factorial analysis of mixed data (FAMD) with a varimax–Kaiser rotation model. *p*-values < 0.05 were considered statistically significant. All analyses were conducted using IBM SPSS Statistics v26 for Windows.

## 3. Results

The socio-demographic variables of the study population are registered in Table 1. An average age of 45.16 ± 10.27 was observed, and represented 56.8% (21/37) of the sample. A total of 70.3% (26/37) of the patients had a body mass index (BMI) > 25 kg/m^2^; 32.4% (12/37) were former smokers, and 40.5% (15/37) are currently employed.

### 3.1. Rheumatological Disease Assessment

Table 2 describes the rheumatological status of SpA patients. Inflammatory back pain was observed in 91.9%, dactylitis in 18.9%, enthesitis in 78.4%, and arthritis in 73.0%. In total, 13.5% of patients presented with CRP > 3 mg/L, and 40.9% presented with HLA B* 27 05 positivity. With regard to clinical indexes, BASDAI and BASFI > 4.0 was observed in more than 50% of the patients, while more than 70% showed mild/moderate activity in the ASDAS-CRP index. At the moment of patient enrollment, the subjects were undergoing treatment as follows: 19.5% were using Non-steroidal anti-inflammatory drugs (NSAIDs), with none presenting with increased calprotectin levels; 4.7% had previously undergone treatment with methotrexate; and 8.2% were given sulfasalazine. The data indicated that 67.6% of subjects were partaking in biological therapy, which included 88.0% utilizing TNF antagonists and 12.0% receiving IL-17 inhibitors, specifically secukinumab. Furthermore, within the TNF inhibitors, the subsequent drugs stood out as particularly important: golimumab (14.6%), adalimumab (24.4%), and certolizumab (7.3%), whereas etanercept was employed in 9.3% of situations; none of the patients were undergoing infliximab treatment.

### 3.2. Gastrointestinal Assessment

The gastrointestinal variables are summarized in Table 3. Among the total patients recruited for this study, 42.2% (76/180) exhibited more than two gastrointestinal symptoms. Of these, 20.6% (37/180) required chromoendoscopy/digital colonoscopy and subsequent histological analysis.

Within this subgroup, 27.0% (10/37) demonstrated elevated FC levels greater than 120 ng/mL PFC, while 16.2% (12/76) exhibited HFC levels exceeding 250 ng/mL. The most prevalent gastrointestinal symptoms reported in this population included abdominal pain and/or bloating in 86.5% (32/37), diarrhea lasting more than four weeks in 59.5% (22/37), and various forms of food intolerance in 62.2% (23/37). Furthermore, 97.3% of the patients identified as omnivorous; among these, 37.8% (14/37) reported intolerance to milk and its derivatives, 18.9% (7/37) to grains, and 13.5% (5/37) to meats

### 3.3. The Macro and Microscopic Evaluation by Colonoscopy

In 21.6% of SpA patients, alterations in the rectal mucosa were observed, with a predominance of loss of the vascular pattern (LVP) in 21.6% of the evaluations and macroscopic features of inflammation in 18.9%. In total, 18.9% of patients presented alterations in the colon mucosa, where the most important finding was a LVP in 10.8%. A total of 45.9% of the patients evaluated showed predominant alterations in the ileum mucosa, whereas 20.7% showed LVP in the ileum (LVPI); 10.8% presented erythema, erosions, and/or ulcers in this region of the small intestine, and 40.5% of the patients showed villous atrophy (VA). Additionally, 27.0% of the patients had hemorrhoids (see Table 4). At the microscopic level, the prevalence of alterations in the intestinal mucosa increased significantly in relation to the macroscopic evaluation (70.3%), with the ileum being the most affected intestinal region, where 40.5% of patients were observed to possess a predominantly inflammatory pattern of inflammation. This was chronic in 32.4% of cases. Architectural alteration was also observed in 32.4% of patients, and cryptitis or VA in 27.0% (see Table 5).

### 3.4. Fecal Calprotectin Associations and FAMD

The bivariate analysis using Pearson’s chi-squared test and/or Fisher’s exact test showed that HFC levels were associated with BASFI > 4 (*p* = 0.036) and BASDAI > 4 (*p* = 0.047), with 70% of these patients presenting LVPI (*p* = 0.005). Furthermore, 77% of the patients who presented PFC with abdominal bloating simultaneously showed LVPI (*p* = 0.020), with 66.7% with VA and microscopic inflammation (*p* = 0.012). Similarly, 75% of those who presented PFC as a whole with abdominal pain also presented LVPI (*p* = 0.007). Surprisingly, 16.1% of the patients presented levels above 250 ng/mL of FC, which were associated with alterations at the level of the ileum mucosa in 35.3% of the patients (*p* = 0.009), and specifically with LVPI in 54.5% (*p* = 0.001).

On the other hand, at the microscopic analysis level, these HFC levels were associated with the presence of inflammation (*p* = 0.046) with a chronic pattern (*p* = 0.014) in 83.3% of patients. Overall, 27.3% of patients presented HFC and diarrhea, associated with LVPI and presence of erosions in the ileum (*p* = 0.026, 0.031). In the same way, microscopically, chronic inflammation was shown to occur in the ileum in 25% of the patients (*p* = 0.037). All patients who presented HFC and ASDASPCR > 2.1 presented alteration of the ileum mucosa (*p* = 0.009), with LVPI (*p* = 0.001) and, in 66.7%, VA, reflected microscopically as a chronic pattern (*p* = 0.014).

To confirm the latest results with a more accurate method, a FAMD was performed. This technique combines principal component analysis (PCA) for continuous variables and multiple correspondence analysis (MCA) for categorical variables, usually in two or more dimensions. It is designed to highlight variations and reveal strong patterns within a dataset, making it easier to explore and visualize. The method employs geometric principles to represent information analogically, studying relationships between variables using canonical Euclidean distance [28]. FAMD allows for the grouping of variables with high correlation coefficients (CCs) and helps identify groups of patients with common characteristics.

**Figure 1:**
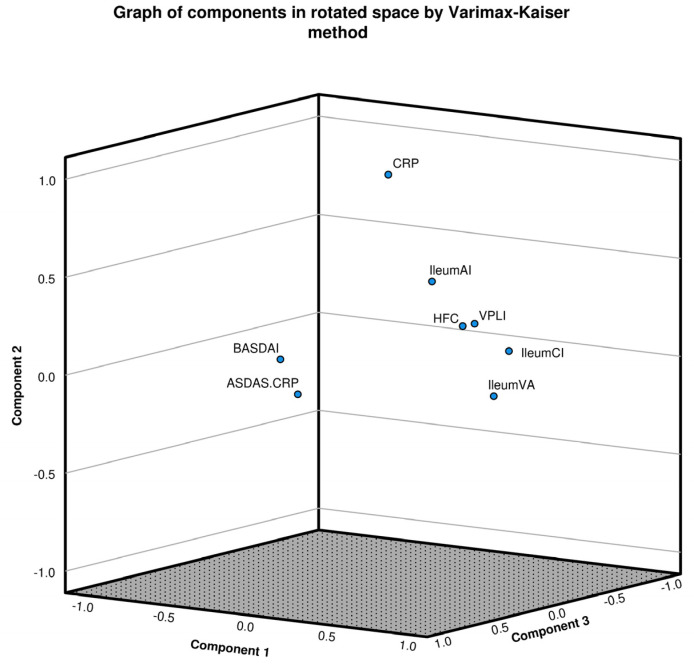
Factorial analysis of mixed data (FAMD) for high levels of calprotectin, activity indices/biomarkers and microscopic changes. FAMD employs principal component analysis (PCA) and multiple correspondence analysis (MCA) across at least two dimensions. This analysis allows for the grouping of variables with high correlation coefficients (CCs), thereby facilitating the differentiation of patient groups with shared characteristics. Each dimension is represented by a correlation coefficient (CC) from each rotated component (dimension) and a CC of the variable within the group, which ranges from −1.0 to +1.0. A high contribution was considered when CC values were >0.7, intermediate when 0.5–0.7, and values between 0.3 and 0.5 were considered low. All values < 0.3 were not included. The Kaiser-Meyer-Olkin (KMO) Test, which measures sampling adequacy for each variable in the model and values > 0.6, indicated that the sampling was adequate, and Bartlett’s test of sphericity was used to evaluate whether there was a redundancy among the variables that we could summarize with fewer factors, where *p* values below 0.05 are accepted. In this model, the KMO test result was 0.642, and the Bartlett test *p* = 0.0001, showed three principal factors that contributed. Dim 1 showed a global CC = 0.837 and grouped micro/macroscopic changes associated with chronic inflammation: vascular pattern loss in ileum (CC 0.772), VA in ileum (0.780), microscopic chronic infiltration of the ileum (0.773) and HFC (0.678), all of them with a global dimension CC of 0.837. Dim 2 showed a global CC of 0.792 and was represented by levels of CRP > 3.0 mg/L (0.841) and microscopic acute inflammation (CC = 0.7728). Finally, Dim 3 showed a global CC of 0.914, grouping activity scores ASDAS-CRP > 2.1 (0.876) and BASDAI > 4.0 (0.812). In spite of HFC’s inclusion in Dim 1, this variable had a CC of 0.332 in the Dim 2, which was graphically observable.

Each dimension is represented by a correlation coefficient CC from each rotated component (dimension) and a CC of the variable within the group, which ranges from −1.0 to +1.0. A high contribution was considered when CC values were >0.7, intermediate when between 0.5 and 0.7, and values between 0.3 and 0.5 were considered low. All values < 0.3 were not included. The Kaiser-Meyer-Olkin (KMO) test, which measures sampling adequacy for each variable in the model and values > 0.6, indicated that the sampling was adequate. On the other hand, Bartlett’s test of sphericity was used to evaluate if there was a redundancy among the variables that we could summarize with a reduced number of factors, where *p* values below 0.05 are accepted. The analysis revealed three principal factors with the following contributions: (1) Micro/macroscopic changes related to chronic inflammation and high-frequency CRP, with a correlation coefficient (CC) of 0.837; (2) high levels of CRP and microscopic acute inflammation, with a CC of 0.792; and (3) clinical activity scores ASDAS-CRP/BASDAI, with a CC of 0.914. This was supported by a Bartlett test with *p* = 0.0001 (see Figure 1).

## 4. Discussion

The gastrointestinal system represents the most extensive mucosal system within the human body, functioning as a primary immunological barrier that safeguards against external pathogens. Consequently, the preservation of the intestinal barrier’s integrity is crucial for sustaining equilibrium and immunological tolerance amid the resident and external microbiota, while governing numerous physicochemical, immunological, and microbiological processes to avert the disruption of homeostasis. When this immunological equilibrium is disrupted, it results in a condition characterized by impaired barrier functions, which facilitates the occurrence of mucosal damage, heightened intestinal permeability, excessive growth of pathogens, and the onset of a proinflammatory state, thereby precipitating bacterial translocation, the migration of microorganisms, and an immune response in organs distant from the intestinal tract [29,30,31].

The established relationship between the gut and joints, which has been recognized for a considerable length time, stems from shared pro-inflammatory pathways, corroborated by clinical research that indicates that 5 to 10% of individuals with AS also face Crohn’s disease or ulcerative colitis [31,32]. Subclinical intestinal inflammation has been detected (through ileocolonoscopy) in 25–49% of individuals afflicted with AS, and histopathological abnormalities within the intestine have been recognized in 50–60% of these patients, implying that there may be a disruption in intestinal permeability and the vascular system, thereby rendering the association between these two conditions conceivable [31]. This evidence implies that a dysfunction in the interface between the gastrointestinal tract and the systemic circulation may play a role in the etiology of these pathological conditions [29]. In the current investigation, we established that 42.2% of the subjects exhibited more than two gastrointestinal manifestations, while 27.0% demonstrated levels of PFC and HFC exceeding 250 ng/mL (16.2%), thereby signifying subclinical indications of neutrophilic degranulation at the intestinal level.

Among the most common gastrointestinal manifestations identified in this investigation, it was noted that 86.5% of the subjects exhibited pain and/or abdominal distension, while 59.5% reported experiencing diarrhea persisting for a duration exceeding four weeks. A variety of macroscopic alterations of the mucosal surface were recorded during the endoscopic examination, including LVPI, the presence of ulcers, and the occurrence of erosive lesions, which were found to correlate with the microscopic histopathological findings, effectively illustrating this association and categorizing them as indicators of potential severity.

Gastrointestinal manifestations concomitant with SpA exhibit an incidence rate ranging from 21% to 30% and encompass a broad clinical spectrum. Approximately 5% to 10% of these manifestations are associated with gastrointestinal disorders [33]. The disease activity indices utilized in SpA do not account for the gastrointestinal condition of the patients. These findings suggest that Colombian patients with active SpA demonstrate a markedly increased prevalence of gastrointestinal manifestations when compared to their counterparts with stable (non-active) disease, as evaluated by the BASDAI and ASDAS indices. As highlighted by the investigations from Romero-Sanchez C [18] and Salas-Cuestas F [34], it has become clear that around 60% of individuals diagnosed with SpA report having gastrointestinal symptoms. Previous investigations have identified that the predominant complaints encompass abdominal distension, abdominal discomfort, and the occurrence of more than two bowel movements within a single day [18,34]. Furthermore, distinctions in the way symptoms are presented were identified across several SpA subtypes (data not revealed), with 62.2% of sufferers reporting food intolerances.

Villous atrophy is characterized by the diminishment or flattening of the intestinal villi, representing a hallmark lesion associated with celiac disease. Nonetheless, this condition is not exclusively restricted to celiac disease, as it has been extensively documented in chronic inflammatory disorders such as IBD [35]. The identification of VA can be accomplished through histopathological examination or via magnified endoscopic assessments, such as those conducted in the current investigation. Furthermore, a thorough evaluation of the vascular architecture and cryptic formation, both of which exhibit degeneration in chronic intestinal inflammatory conditions, can be facilitated through chromoendoscopy. Various research efforts have validated the existence of these histological changes in individuals with a SpA diagnosis [16,36,37,38,39]. One particular investigation has recorded various patterns of intestinal alterations characterized by acute inflammation resembling bacterial enterocolitis, and persistent inflammation similar to Crohn’s disease, noted for distortion of the crypts alongside VA, flattening, and fusion of the villi, accompanied by significant infiltration of mononuclear cells within the lamina. Specific histological modifications within the intestines of individuals with SpA, irrespective of the extent of intestinal inflammation, have also been documented [36]. These modifications encompass hyperplasia of goblet cells, augmented mucin production, and the activation of Paneth cells [36]. This investigation found that a notable 21.6% of patients diagnosed with SpA demonstrated macroscopic indications of inflammation in their rectal mucosa, alongside a marked LVP in 21.6% of instances. Concurrently, LVP was identified in 10.8% of colon biopsies, while 45.9% of the evaluated patients predominantly exhibited mucosal alterations within the ileum, wherein 20.7% displayed LVPI.

Multiple inquiries have found that FC could be a significant distinguishing marker between functional and inflammatory gastrointestinal disorders, thus promoting the monitoring of intestinal inflammatory activity after a diagnosis of IBD is made, in addition to gauging treatment success by examining mucosal healing and estimating relapse potential [13,15,38,39]. This study uncovered significant correlations between elevated FC levels and increased activity scores, with 70% of the subjects demonstrating notable LVPI. In this population of SpA patients with gastrointestinal inflammation, FC possesses the potential to be recognized as a crucial non-invasive and sensitive biomarker for the evaluation of intestinal inflammation associated with these conditions.

Studies have shown that FC levels are significantly heightened in patients diagnosed with SpA, even in the absence of IBD [13,38,39,40,41,42,43,44]. Olofsson T et al. (2019) observed that elevated FC levels in individuals with ax-SpA are associated with intensified joint disease activity and reduced physical functionality. They posited that this biomarker serves as a critical indicator of more advanced disease manifestations [38]. Klingberg E et al. (2012) conducted an evaluation of fecal calprotectin (FC) levels within a cohort comprising 205 individuals diagnosed with ankylosing spondylitis (AS); their findings revealed that 68% of the subjects exhibited elevated FC levels (greater than 50 mg/kg) and that FC levels were positively correlated with heightened disease activity, although no association was observed with gastrointestinal manifestations [42].,

In addition, Klingberg et al. (2017) undertook a longitudinal investigation over five years with a cohort of AS patients who presented with gastrointestinal symptoms; they highlighted the essential importance of quantifying FC particularly in individuals exhibiting elevated disease activity in conjunction with gastrointestinal manifestations, demonstrating that these levels were consistently high in the majority of patients and correlated with disease activity [13]. This investigation delineates that the prevalence of Crohn’s disease was observed to be 1.5% among individuals diagnosed with AS, particularly those exhibiting elevated FC levels, thereby indicating that this molecule may function as a biomarker for the identification of AS patients who are predisposed to the development of IBD [13]. The detection of elevated FC emerged as a key indicator for AS, thereby underscoring the link between gut inflammation and musculoskeletal symptoms in AS, and implying that biomarker might function as a potential biomarker for recognizing AS patients who may develop IBD [13,37,38,41,42,43]. In the study of Klingberg et al. (2017), FC was higher in patients treated with NSAIDs and proton pump inhibitors, and lower in patients treated with a TNF blocker or methotrexate [13]. However, our results showed that none of the HFC patients were under NSAIDs treatment. 

This investigation substantiates that 27.0% of individuals exhibited elevated FC levels, with HFC observed in 16.2%, revealing significant correlations between this biomarker and elevated BASFI and BASDAI scores. Furthermore, 16.1% of patients HFC levels showed a significant correlation with mucosal changes in the ileum (35.3%, *p* = 0.009), particularly with LVPI alterations, observed in 54.5% of cases (*p* = 0.001). According to Rizzo et al. (2018), subclinical intestinal inflammation in SpA could be seen as an essential trigger for the disease, since the dynamics between the microbiome and the host immune system can compromise intestinal barriers and eventually activate inflammatory responses in regions beyond the intestine, which could incite chronic inflammation in SpA [36].

Although similar investigations have been conducted, none have incorporated the utilization of digital chromoendoscopy, which enhances the capacity to identify subtle alterations in intestinal mucosal integrity that would likely remain undetected through traditional colonoscopy, such as LVPI. In addition, this study also allowed us to correlate FC levels with such changes in SpA patients without IBD. The results revealed that 70.0% of patients presented LVPI, which correlated with abdominal symptoms and microscopic inflammation and showed a significant correlation between elevated levels of FC, LVPI, ileal VA, and activity and function indices in SpA patients without IBD, indicating a shared immunological pathway of joint activity linked to chronic intestinal damage. These findings have not been previously described, suggesting that subclinical intestinal inflammation may trigger disease activity. These results support that FC may be a noninvasive biomarker of very early changes in the intestinal mucosa of SpA patients that could be valuable for monitoring intestinal inflammation in SpA patients, differentiating it from functional gastrointestinal disorders, which have not been emphasized in previous research.

## 5. Limitations

The primary constraint of this investigation is that the size of the sample analyzed was notably limited; although the study commenced with a considerable cohort of patients diagnosed with SpA (*n* = 180), only 20.6% (37/180) satisfied the stringent inclusion criteria specifically requiring a colonoscopy assessment and a comprehensive histological examination. A larger sample size could potentially lead to greater statistical validity. However, despite this limitation, the research provides important contributions towards the early detection of FC levels in SpA patients who do not simultaneously demonstrate IBD, and demonstrates a strong relationship between increased levels of this biomarker and parameters of disease activity and functionality, together with both macro and microscopic findings at the intestinal level. The clinical relevance of the findings remains substantial, particularly considering the difficulties encountered in identifying subclinical inflammation within this population, which demands further research involving larger sample sizes and longitudinal assessments to reinforce the evidence delineated herein, with the objective of promoting the future use of FC as a biomarker reflective of subclinical intestinal inflammation in SpA and/or as a prognostic marker for the potential onset of IBD in the imminent future.

The investigation primarily focused on individuals who have been diagnosed with SpA, as well as on the conventional diagnostic methodologies, including colonoscopy and histological sampling, which are typically not performed on healthy individuals or on SpA patients who do not present gastrointestinal (GI) symptoms. Consistent with ethical principles, it would be considered unethical to expose healthy individuals or those devoid of GI symptoms to invasive techniques solely for research aims. Thus, our research was limited to the demographic of patients displaying GI symptoms, a frequent complication noted in investigations that utilize such invasive diagnostic approaches.

## 6. Conclusions

The results support that SpA patients who exhibit high levels of FC show LVPI as a main chromo-colonoscopy finding, which is associated with disease activity score. Micro/macroscopic inflammatory changes in the ileum suggest an immune connection with joint manifestations. The FC results are promising and merit further investigation. Further studies on FC evaluation and its relationship with gastrointestinal conditions in SpA patients are needed to validate this molecule as a noninvasive biomarker.

## Figures and Tables

**Table 1:** Sociodemographic status of patients with SpA evaluated by colonoscopy.

	SpA *n* = 37	
Age *	45.2 ± 10.3	
BMI **	25.9 (24.8–28.4)	
	*n*	%
Sex		
Male	21	56.8
BMI > 25	26	70.3
Current smoker	2	5.4
Former smoker	12	32.4
Passive smoker	8	21.6
Economy activity		
Home	6	16.2
Independent	9	24.3
Employee	15	40.5
Pensioner	5	13.5
Student	2	5.4
Marital status		
Married	20	54.1
Single	9	24.3
Common-law marriage	8	21.6
Education level		
Primary	1	2.7
High school	12	32.4
Technician	5	13.5
University	19	51.4

* Results are expressed in means ± standard deviation. ** Results are expressed in medians (IQR). BMI: body mass index.

**Table 2:** Rheumatological status of SpA patients.

	SpA *n* = 37	
	*n*	%
ASAS criteria		
Axial	33	89.2
Peripheral	4	10.8
SpA symptoms		
Inflammatory low back pain	34	91.9
Mechanical low back pain	7	18.9
Dactylitis	7	18.9
Enthesitis	29	78.4
Arthritis	27	73.0
Fatigue	30	81.1
Treatment		
Biological	25	67.6
Type of treatment		
Anti-IL17	3	12.0
Anti-TNF	22	88.0
CRP **	0.78 (0.28–2.24)	
>3 mg/L	5	13.5
ESR	4.5 (2.6–7.3)	
Faster-than-normal rate	20	54.1
HLA B27 positive	15	40.5
BASFI **	5.0 (3.2–7.1)	
BASFI > 4	22	59.5
BASDAI **	2.6 (2.2–3.0)	
BASDAI > 4	29	78.4
ASDAS-CRP **	2.6 (2.2–3.0)	
ASDAS-CRP > 2.1	29	78.4
ASDAS-CRP > 3.5	5	13.5
ASDAS-ESR **	2.8 (2.3–3.5)	
ASDAS-ESR > 2.1	31	83.8
ASDAS-ESR > 3.5	10	27.0

** Results are expressed in medians (IQR). CRP: C-reactive protein; ESR: erythrocyte sedimentation rate; BASDAI: Bath Ankylosing Spondylitis Disease Activity Index; BASFI: Bath Ankylosing Spondylitis Functional Index; ASDAS: Ankylosing Spondylitis Disease Activity Score; HLA: human leukocyte antigen.

**Table 3:** Gastrointestinal status of SpA patients.

	SpA *n* = 37	
	*n*	%
Calprotectin **	64.6 (44.29–138.41)	
>120 ng/mL	10	27.0
Type of feeding		
Omnivorous	36	97.3
>2 gastrointestinal symptoms	37	100.0
>4 weeks diarrhea	22	59.5
Blood in stool	7	18.9
Mucus in stool	11	29.7
Abdominal pain	32	86.5
Weight loss	10	27.0
Abdominal distension	32	86.5
Food intolerance	23	62.2
Fruits	1	2.7
Vegetables	2	5.4
Grains	7	18.9
Meat	5	13.5
Milk and derivatives	14	37.8
Fat and oils	2	5.4
Others	3	8.1
Non identifiable food	2	5.4

** Results are expressed in medians (IQR).

**Table 4:** Macroscopic assessment of SpA patients by digital chromo-endoscopy (DCE).

	SpA *n* = 37	
	*n*	%
Rectus		
Affected mucous	8	21.6
Erythema	2	5.4
Vascular pattern loss	8	21.6
Erosions	4	10.8
Ulcers	1	2.7
Inflammation	7	18.9
Sigmoid colon		
Affected mucous	7	18.9
Erythema	1	2.7
Vascular pattern loss	4	10.8
Erosions	3	8.1
Ulcers	0	0.00
Ileum		
Affected mucous	17	45.9
Erythema	4	10.8
Vascular pattern loss	11	20.7
Erosions	4	10.8
Ulcers	4	10.8
Atrophy of intestinal villous	15	40.5
Hemorrhoids	10	27.0

**Table 5:** Histological assessment of SpA patients.

	SpA *n* = 37	
	*n*	%
Histological alteration	26	70.3
Ileum		
Inflammatory pattern	15	40.5
Architecture alteration	12	32.4
Cryptitis/atrophy	10	27.0
Chronic inflammation	12	32.4
Acute inflammation	5	13.5
Submucous compromise	1	2.7
Colon		
Inflammatory pattern	12	32.4
Cryptitis/atrophy	6	16.2
Chronic inflammation	10	27.0
Acute inflammation	7	18.9
Submucous compromise	1	2.7
Rectus		
Inflammation	8	21.6
Eosinophilia	2	5.4

## Data Availability

The data used to support the findings of this study are available from the corresponding author upon request.
